# Regulatory Function of Sympathetic Innervation on the Endo/Lysosomal Trafficking of Acetylcholine Receptor

**DOI:** 10.3389/fphys.2021.626707

**Published:** 2021-03-11

**Authors:** Tatjana Straka, Charlotte Schröder, Andreas Roos, Laxmikanth Kollipara, Albert Sickmann, Marion Patrick Ivey Williams, Mathias Hafner, Muzamil Majid Khan, Rüdiger Rudolf

**Affiliations:** ^1^Institute of Molecular and Cell Biology, Mannheim University of Applied Sciences, Mannheim, Germany; ^2^Institute of Toxicology and Genetics, Karlsruhe Institute of Technology, Karlsruhe, Germany; ^3^Interdisciplinary Center for Neurosciences, Heidelberg University, Heidelberg, Germany; ^4^Leibniz-Institut für Analytische Wissenschaften—ISAS—e.V., Dortmund, Germany; ^5^Department of Neuropediatrics, University Hospital Essen, Essen, Germany; ^6^Children’s Hospital of Eastern Ontario Research Institute, Ottawa, ON, Canada; ^7^Department of Chemistry, College of Physical Sciences, University of Aberdeen, Aberdeen, United Kingdom; ^8^Medizinische Fakultät, Medizinische Proteom-Center (MPC), Ruhr-Universität Bochum, Bochum, Germany

**Keywords:** sympathectomy, sympathetic nervous system, neuromuscular junction, skeletal muscle, endo/lysosomal trafficking, acetylcholine receptor

## Abstract

Recent studies have demonstrated that neuromuscular junctions are co-innervated by sympathetic neurons. This co-innervation has been shown to be crucial for neuromuscular junction morphology and functional maintenance. To improve our understanding of how sympathetic innervation affects nerve–muscle synapse homeostasis, we here used *in vivo* imaging, proteomic, biochemical, and microscopic approaches to compare normal and sympathectomized mouse hindlimb muscles. Live confocal microscopy revealed reduced fiber diameters, enhanced acetylcholine receptor turnover, and increased amounts of endo/lysosomal acetylcholine-receptor-bearing vesicles. Proteomics analysis of sympathectomized skeletal muscles showed that besides massive changes in mitochondrial, sarcomeric, and ribosomal proteins, the relative abundance of vesicular trafficking markers was affected by sympathectomy. Immunofluorescence and Western blot approaches corroborated these findings and, in addition, suggested local upregulation and enrichment of endo/lysosomal progression and autophagy markers, Rab 7 and p62, at the sarcomeric regions of muscle fibers and neuromuscular junctions. In summary, these data give novel insights into the relevance of sympathetic innervation for the homeostasis of muscle and neuromuscular junctions. They are consistent with an upregulation of endocytic and autophagic trafficking at the whole muscle level and at the neuromuscular junction.

## Introduction

Endocytosis of signaling transmembrane proteins, such as EGFR (Caldieri et al., 2018), CD81 (Hosokawa et al., 2020), NMDA-R (Scott et al., 2004), AMPA-R (Ehlers, 2000), or ion channels in general (Estadella et al., 2020), is initiated by Clathrin-dependent or -independent invagination of plasma membrane, resulting in transmembrane receptor-containing early endosomes. Trafficking and maturation of endosomes are regulated by Rab GTPases, which are small GTP-binding proteins. Specific Rab proteins are associated with certain stages of endocytosis. In particular, very early endosomal processing is linked to Rab 5, with its presence found to be rate limiting in the endocytic pathway (Bucci et al., 1992). Upon endosomal maturation, Rab 5 is exchanged by Rab 7 to reach a state of late endosomes. They are delivered to lysosomal degradation either directly (Chavrier et al., 1990; Soldati et al., 1995) or via a process known as macroautophagy (hereafter short autophagy) (Amaya et al., 2015). Autophagy is a specialized self-eating process of cellular components by delivering them to lysosomes (De Duve, 1963) and provides a degradation route for endocytosed vesicles. Apart from Rab 7, there are other Rab GTPases involved in facilitating the delivery of late endosome to autolysosomes (Amaya et al., 2015). For instance, Rab 1b was required for autophagosome formation from specific sites of the ER (Amaya et al., 2015). Furthermore, while Rab 11 is known to be associated with recycling endosomes and post-Golgi vesicular trafficking (Lock and Stow, 2005; Welz et al., 2014), some studies point toward an additional role of this Rab GTPase in autophagy regulation (Fader et al., 2008; Longatti et al., 2012; Szatmári et al., 2014; Puri et al., 2018).

At the neuromuscular junction (NMJ), the main signaling transmembrane receptor is acetylcholine receptor (AChR). Here, at the interphase between skeletal muscle and motoneuron innervation, a high density of AChR (roughly 10,000 receptors/μm^2^) ensures efficient neuromuscular signal transmission (Sanes and Lichtman, 2001). Remodeling of the NMJ upon disturbances, such as sciatic nerve lesion, leads to reduction in AChR lifetime and fragmented morphology of the band-like appearance of rodent NMJs often described as “pretzel-shaped” (Levitt et al., 1980; Levitt and Salpeter, 1981; Stanley and Drachman, 1981, 1987; Shyng et al., 1991; Strack et al., 2011, 2015; Tu et al., 2017; Vannucci et al., 2019). Changes in AChR localization, lifetime, and/or density are hallmarks of several disease states (Fambrough et al., 1973; Drachman et al., 1980; Webster, 2018), denervation (Levitt et al., 1980; Levitt and Salpeter, 1981; Stanley and Drachman, 1981, 1987; Shyng et al., 1991; Strack et al., 2011, 2015; Tu et al., 2017; Vannucci et al., 2019), and aging (Gonzalez-Freire et al., 2014; Rudolf et al., 2014; Taetzsch and Valdez, 2018). AChR localization, lifetime, and/or density at NMJs are tightly correlated with regulation of endocytosis and vesicle trafficking (reviewed in Rudolf and Straka, 2019). Indeed, overexpression of Rab 5 or its constitutively active mutants led to an increase in AChR-positive endocytic vesicles, suggesting an involvement of Rab 5 in the endocytosis of AChR (Wild et al., 2016). Furthermore, vesicle trafficking of AChR was dependent on the membrane-curvature inducing and endo/autophagosomal regulator protein Endophilin B1 (aka SH3GLB1 or Bif 1). More precisely, phosphorylation of Endophilin B1 by cyclin-dependent kinase 5 (Cdk5) (Wong et al., 2011) was shown to modulate Rab 5 activity. Particularly, overexpression of a dominant-negative mutant of Cdk5 (DN-Cdk5) caused a reduction of denervation-induced endocytic AChR-containing vesicles (Wild et al., 2016). These data are consistent with the observation of a cooperative function of Cdk5, Endophilin B1, and Beclin1 in autophagy (Wong et al., 2011; Ishii et al., 2019). At rodent NMJs, degradation of endocytosed AChR vesicles has been shown to occur via autophagy in an Endophilin B1 and MuRF1-dependent manner (Khan et al., 2014). Here, colocalization of AChR-positive endocytic vesicles and autophagosomal components, like LC3, p62, and MuRF1, was observed upon induction of muscle atrophy (Khan et al., 2014).

In terms of molecular signaling, AChR stability is—among others—regulated by cAMP, protein kinase A (PKA), and α-calcitonin gene-related peptide (Laufer and Changeux, 1987; Miles et al., 1989; Shyng et al., 1991; Poyner, 1992; Lu et al., 1993; Xu and Salpeter, 1997; Röder et al., 2010; Martinez-Pena y Valenzuela et al., 2013; Machado et al., 2019). In this context, close proximity between sympathetic neurons and NMJs (Chan-Palay et al., 1982; Khan et al., 2016; Rodrigues et al., 2018; Straka et al., 2018; Snyder-Warwick et al., 2018), and the relevance of cAMP in G-protein-coupled receptor signaling of adrenergic receptors, pointed to a potential role of sympathetic signaling in regulating AChR endocytic trafficking. Indeed, chemical or surgical sympathectomy led to NMJ shrinkage, and reduced membrane-bound AChR (Khan et al., 2016; Rodrigues et al., 2018). Furthermore, C2C12 myoblasts showed enhanced AChR stabilization upon application of sympathicomimetic α-adrenergic receptor agonists (Clausen et al., 2018), corroborating an involvement of the sympathetic nervous system in AChR turnover. Fittingly, treatment with sympathicomimetics was beneficial in clinical treatment of several congenital myasthenic syndromes caused by inherited mutations of NMJ proteins frequently followed by decreased AChR density (Ohno et al., 2002; Chevessier et al., 2004; Bestue-Cardiel et al., 2005; Lashley et al., 2010; Liewluck et al., 2011; Sadeh et al., 2011; Wargon et al., 2012; Burke et al., 2013; Lorenzoni et al., 2013; Tsao, 2015; Mulroy et al., 2017; Padmanabha et al., 2017). Although changes at the NMJ were observed upon sympathectomy, it remained unclear whether the sympathetic nervous system was involved in the previously described endo/lysosomal trafficking of AChR-containing endosomes. To address this question, we performed proteomic analysis of sympathectomized hind limb muscles. These results are further supported by Western blot and immunofluorescence analysis. Results of these combined biochemical studies revealed a regulatory function of the sympathetic nervous system on the endo/lysosomal pathway, which supports the finding of reduced membrane-bound AChR and NMJ shrinkage upon sympathectomy (Khan et al., 2016; Rodrigues et al., 2018).

## Materials and Methods

### Animals

In the present study, adult C57BL/10J mice were used. Animals were maintained in a local animal facility, and their use, care, and experimental protocols were approved by the commission of ethics in animal research of the national authorities in Germany (Regierungspräsidium Karlsruhe, G-285/14, June 11, 2016 and local ethical committee).

### Antibodies

The following antibodies and dyes were used in the present study (Table 1). Anti-Tyrosine hydroxylase (TH) antibodies were chosen, since postganglionic sympathetic neurons typically release norepinephrine. Therefore, TH, which converts tyrosine into 3,4-dihydroxyphenylalanine (L-DOPA), a precursor of noradrenalin, is frequently used as a marker for sympathetic neurons (Forsgren and Söderberg, 1987; Loesch et al., 2009), also in skeletal muscles (Rodrigues et al., 2018).

**TABLE 1:** Antibodies.

Name	Manufacturer/product number	Whole mount	Western blot	Cryosection
Synaptophysin	SySy/1010044	1:100	−	−
Neurofilament	SySy/171002	1:100	−	−
TH	Merck/ab152	1:50	−	−
CD31	Biotechne/AF3628	1:50	−	−
Rab 5	Cell Signaling/3547	−	1:1,000	−
Rab 7a	SySy/320 003	−	1:1,000	1:200
Rab 1b	Santa Cruz/sc-599	−	1:500	−
Rab 11	Cell Signaling/3539	−	1:1,000	−
Beclin1	Cell Signaling/3495	−	1:1,000	−
NCAM1	Cell Signaling/99746	−	1:1,000	−
Cdk5	Santa Cruz/sc-173	−	1:500	1:200
p62	Progen/GP62-C	−	1:500	1:200
GAPDH	ThermoFisher/MA5-15738	−	1:10,000	−
Anti-rb 647 +	Invitrogen/A32795	1:200	−	1:1,000
Anti-gp 555	Invitrogen/A21435	1:200	−	1:1,000
Anti-gt 546	Invitrogen/A11056	1:200	−	−
BGT 488	Invitrogen/B13422	1:200	−	−
BGT 555	Invitrogen/B35451	−	−	1:1,000
BGT 647	Invitrogen/B35450	1:200	−	1:1,000
Anti-ms HRP	Invitrogen/32430	−	1:10,000	−
Anti-rb HRP	Dianova/111-035-003	−	1:10,000	−
Anti-gp HRP	Thermo Fisher/PA1-28597	−	1:10,000	−
DAPI	SIGMA/10236276001	1:100	−	1:1,000

*Manufacturer and product number of applied antibodies as well as used dilution (rb, rabbit; gp, guinea pig; gt, goat).*

### Clearing, Immunostaining, Imaging, and Data Processing of Diaphragms

Adult diaphragms were chemically fixed in freshly prepared 4% PFA (5 min, RT). For immunostaining, a slightly modified iDISCO-based staining protocol was applied as previously described for P0 and P30 diaphragms (Renier et al., 2014; Straka et al., 2018). In brief, diaphragms were dissected and incubated in blocking and permeabilization solution (BnP) composed of 1 × PBS/1 × PTwH (0.2% tween in 1 × PBS with 10 μg/ml heparin)/0.5% Triton X-100/10% (vol/vol) DMSO/6% (vol/vol) BSA (1 × BnP) for 3 days followed by a 24 h-incubation in quenching solution (1 × PBS/0.5% Triton X-100/20% DMSO/0.3 M glycine). The primary antibody was diluted in 1 × BnP and incubated at 37°C on an orbital shaker for 4 days. Then, diaphragms were washed with 1 × PTwH for 4-5 days and incubated with secondary antibody, nuclear dye DAPI, and BGT (diluted with 1 × BnP) at 37°C for 24 h on an orbital shaker. Before imaging, another 3 days of 1 × PTwH and 24 h of ddH_2_O washing were performed. For BGT-conserving refractive index matching, a gradient of increasing glycerol concentrations (20, 40, 60, 80, and 88% glycerol in ddH_2_O) was performed. Lower glycerol concentrations (20–60%) were incubated for 10 h or until the tissue sunk. Higher concentrations (80 and 88%) were incubated for at least 24 h. Then, diaphragms were mounted on a glass slide embedded in 88% of glycerol. Coverslips were fixed on a glass slide using picodent twinsil (picodent/1,300 1,000). If temperature was not specified, incubation was performed at room temperature. Images were taken using an inverted Leica TCS SP8 microscope equipped with 405, 488, 555, and 633 nm lasers, and Leica HC PL APO CS2 20 × /0.75 IMM CORR objective. Scan settings included 1,024 × 1,024-pixel resolution, 0.75 × zoom, two times line average, pinhole setting of 1 Airy Unit, and bi-directional scan speed of 600 Hz. Voxel size was 0.758 × 0.758 × 4.5 or 5 μm. Z-compensation was applied. Automated tile scan imaging was performed using the Leica LAS X 3.5 navigator module followed by automated stitching (smooth mode). Projections of diaphragms were rendered with the 3D visualization module of LAS X.

### Chemical Sympathectomy

Chemical sympathectomy was induced by the use of 6-hydroxydopamine (6OHD) as previously described (Khan et al., 2016). Briefly, 6OHD (ChemCruz/sc-203482) was diluted in 0.3% ascorbic acid oxygen-free water and injected into the tibialis anterior muscle (100 mg/kg) on alternate days for 2 weeks before tissue extraction. Tibialis anterior was chosen due to its size and position; both these factors make it suitable for frequent injections. In addition, it is a well-studied muscle for live cell imaging (Rudolf et al., 2012). For injection, anesthesia using inhalation of Isoflurane (cp-pharma/AP/DRUGS/220/96) was used. Control animals received intramuscular 1 × PBS injection. Right after tissue extraction, the treated muscle was cut longitudinally in two halves. One half was further processed for Western blot, whereas the other half was used in proteomic or cryosection analysis.

### *In vivo* Visualization and Measurement of Fiber Diameter, Acetylcholine Receptor Turnover Rate, and Vesicle Numbers

AChR turnover was measured as described previously (Röder et al., 2010; Choi et al., 2012; Khan et al., 2014). In brief, BGT 647 and BGT 555 (25 pmol each) were sequentially injected into tibialis anterior muscles at a temporal distance of 10 days. After the second injection, the upper 200 μm of these muscles were examined *in vivo* with an upright Leica SP2 (Leica Microsystems) confocal microscope using a 63 × /1.2 NA water immersion objective. For the analysis of AChR turnover, 3D stacks at 512 × 512-pixel resolution were taken of BGT 647 (“old AChR”) and of BGT 555 signals (“new AChR”). From these images, fiber diameters were determined taking advantage of the slight BGT fluorescence along the sarcolemma. Therefore, for each fiber, the maximal width in the image stack was determined and measured with ImageJ. AChR turnover rate was defined by the ratio of “new AChR” and “old AChR” mean signal intensity at the NMJ. Therefore, NMJ ROIs were hand segmented in the BGT 647 channel, and the mean intensity within this ROI was measured in both channels. The number of BGT-positive vesicles was done by segmenting dot-like fluorescent structures in the BGT 647 channel that were at or close around the NMJs.

### Proteomics

For a detailed instruction of proteomic material and methods, see Supplementary Document 1. The volcano plot in the proteomics section was created using Microsoft Excel. Generation of the proteomics heatmaps used the open-source software Perseus 1.6.2.3^1^.

### Western Blot

For Western blot analysis, half tibialis anterior muscles were snap-frozen, lysed using lysis buffer [50 mM Tris-HCl pH 7.8, 150 mM NaCl, 1% NP-40 (AppliChem/A1694), 10% glycerol, 5 mM EDTA, 1 mM EGTA, 1 Halt Protease and Phosphatase Single-Use Inhibitor Cocktail (FisherScientific/10025743), and 0.5 mM PMSF (AppliChem/A0999), pH adjusted to 7.4], and subjected to SDS-PAGE followed by Western blot analysis as already described (Wild et al., 2016; Straka et al., 2018). In each lane, equal amounts of protein were loaded (20 μg). Chemiluminescence signals were obtained using an ECL system (Biozym Scientific GmbH/541004) in combination with a Syngene G:Box Chemi XX6 chemiluminescence imager (Thermo Fisher Scientific, Schwerte, Germany). The analysis used ImageJ freeware image processing software^2^.

### Immunostaining, Imaging, and Data Processing of Muscle Cryosections

For immunofluorescence of cryosections, half tibialis anterior muscles were embedded in FSC 22 Clear (3801480; Leica Biosystems Nussloch GmbH, Germany), frozen over liquid nitrogen, and cut in 10 μm-thick slices using Leica Cryostat CM1950 (Leica Microsystems, Wetzlar, Germany). Sections were quickly washed with 1 × PBS, permeabilized with 0.1% Triton-X100/PBS (10 min), washed with 1 × PBS (2 × 5 min), washed with 2% BSA/PBS (5 min), and blocked with 2% BSA/PBS (2 h, 4°C). Then, sections were incubated with primary antibodies in 2% BSA/PBS (overnight, 4°C). After washing with 2% BSA/PBS (3 × 5 min), the slides were incubated with secondary antibodies and BGT in 2% BSA/PBS (3 h, RT, dark) followed by washing with 2% BSA/PBS (2 × 5 min). Nuclei were stained using DAPI in 2% BSA/PBS (5 min), followed by 2 × 5 min washes with 2% BSA/PBS and 2 × 5 min washes with ddH_2_O. Slides were embedded in Mowiol. Images were acquired using the same microscope and settings as previously described for diaphragm; see above. Image processing and analysis were conducted with ImageJ and Microsoft Excel. Therefore, all (including weak) NMJs were segmented using the BGT staining and variable thresholding creating regions of interest (ROIs). Within these ROIs, the mean intensity was measured in either the same channel (BGT staining) or other channels (Rab 7a, p62, Cdk5 staining). Similarly, muscle fiber mean intensity was evaluated. Therefore, an ROI spanning the muscle fiber was drawn, and signal mean intensity within this ROI was measured in the antibody channels (Rab 7a, p62, and Cdk5). Additionally, the fiber size was taken from this ROI. The mean intensity was normalized to intensity at NMJs or MF of PBS sections processed together with 6OHD-treated sections.

### Statistical Analysis

Statistical analyses were performed using Microsoft Excel, and Student’s *t*-test (two tailed, unpaired) were performed to establish *P*-values. Values are reported as means ± SEM or means ± STD. Please refer to the figure legends for more information.

## Results

### Sympathetic Innervation in Whole Mount Adult Diaphragm

**FIGURE 1:**
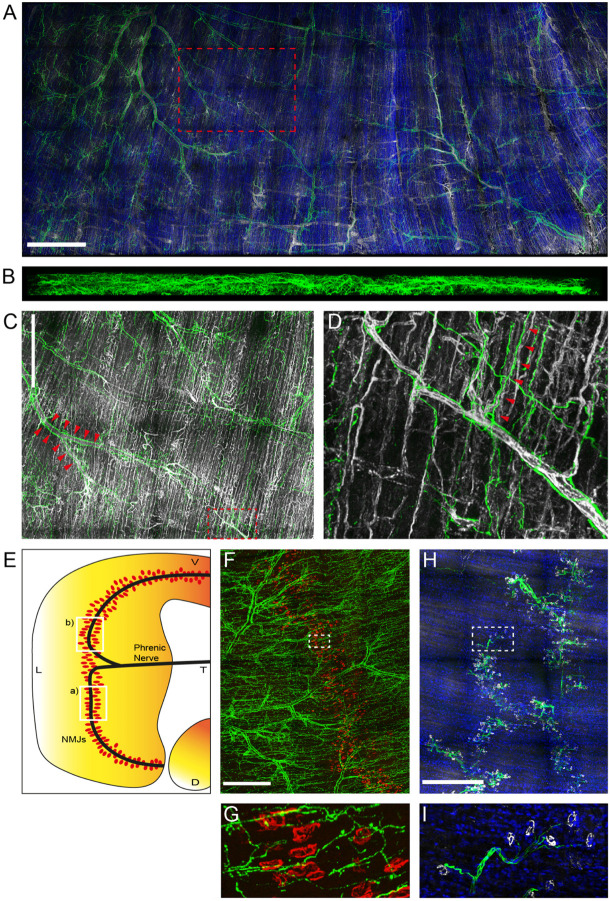
Sympathetic innervation is intense in adult mouse diaphragm muscle. Diaphragm muscles from 12 to 19 weeks old mice were fixed and stained with antibodies against TH (green), CD31 (white), nuclei (DAPI, blue) **(A–D)**, against anti-TH (green), and BGT 555 (red) **(F,G)**, or against anti-neurofilament (green), anti-synaptophysin (white), and 4′,6-diamidino-2-phenylindole [DAPI (blue)] **(H,I)**. Then, muscles were imaged by tile scanning confocal microscopy with a voxel size of 0.758 × 0.758 × 5 μm. Maximum z-projections were rendered using the 3D visualization module of LAS X. For clarity, DAPI labels were omitted in **(B–D)** and, additionally, CD31 signals were left out in **(B)**. Boxed regions are shown at higher magnification in the following order: box A shown in **(C)**, box C shown in **(D)**, box F shown in **(G)**, box H shown in **(I)**. Arrowheads in **(C,D)** indicate ramifications of sympathetic neurons initiating from major branches of blood vessels **(C)** and running along muscle fibers and small blood vessels **(D)**, respectively. Scale bars represent **(A)** 1 mm, **(C,F,H)** 500 μm. **(E)** Schematic indicating the approximate diaphragm portions shown in **(F,H)** as boxed regions a) and b), respectively.

Previously, the sympathetic neural network of P0 and P30 mice was examined and an age-related increase in innervation complexity observed (Straka et al., 2018). Using a slightly adapted experimental protocol, staining and imaging of diaphragms from 12 to 19 weeks old adult mice has now been achieved. Figure 1A shows a maximum z-projection of a large region of an adult diaphragm stained with antibodies against tyrosine hydroxylase (TH; rb anti-TH labeled with anti-rb AlexaFluor647, green) and endothelial cell adhesion molecule CD31 (CD31; gt anti-CD31 labeled with anti-gt AlexaFluor546, white). These are markers for sympathetic neurons and blood vessels, respectively. In addition, cell nuclei were labeled with 4′,6-diamidino-2-phenylindole (DAPI, blue). This corroborated the well-known association of sympathetic neurons with large blood vessels (reviewed in Di Bona et al., 2019). Figure 1B depicts a side view of the same diaphragm. For clarity, nuclear and CD31 signals were omitted here. This image demonstrates that the entire diaphragm with a thickness of roughly 500 μm was well penetrated by the light microscope and that the sympathetic innervation was present throughout. Further, concentration on details demonstrated ample ramifications of sympathetic neurons that were often found initiating from major branches of blood vessels (Figure 1C; shown region is boxed in Figure 1A; exemplary sympathetic ramifications branching from large blood vessels, see arrowheads) and then ran along muscle fibers and small blood vessels (Figure 1D; shown region is boxed in Figure 1C; exemplary sympathetic fibers running along small blood vessels, see arrowheads).

Next, the correlation between NMJs and sympathetic innervation was addressed in different diaphragm regions, as indicated in Figure 1E, boxes labeled (a) and (b). The first region was stained with rb anti-TH antibody and anti-rb AlexaFluor647 (green, Figures 1F,G) and fluorescent α-bungarotoxin AlexaFluor 555 (BGT 555; red, Figures 1F,G). BGT is a marker of nicotinic AChR. This confirmed a close distance between NMJs and sympathetic neurons, but showed no plaque-like enrichment of anti-TH signals at the NMJ region, as previously observed for some hindleg muscles (Straka et al., 2018). In contrast to the highly branched sympathetic innervation pattern, motoneurons, stained with antibodies against neurofilament (rb anti-neurofilament labeled with anti-rb AlexaFluor488; green, Figures 1H,I) and synaptophysin (gp anti-synaptophysin labeled with anti-gp AlexaFluor555; white, Figures 1H,I), traversed the muscle in nerve bundles to reach the synapse band, roughly in the central part of the muscle.

### Chemical Sympathectomy Increases Acetylcholine Receptor Turnover and Number of Endo/Lysosomal AChR Vesicles

**FIGURE 2:**
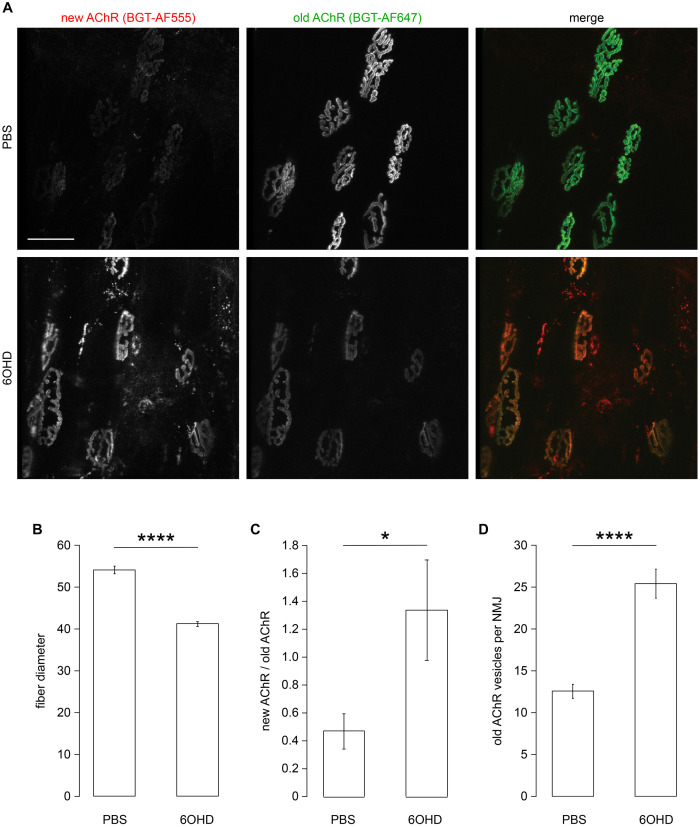
Sympathectomy affects muscle fiber diameter, acetylcholine receptor (AChR) turnover, and number of endo/lysosomal BGT-positive vesicles. Tibialis anterior muscles were injected with either PBS or 6-hydroxydopamine (6OHD) for 2 weeks on alternate days. Four days after the start of these treatments, the muscles were injected with BGT 647. At the end of PBS/6OHD treatment, the muscles were prepared for visual inspection, injected with BGT 555, and then analyzed by live confocal microscopy. **(A)** Representative maximum z-projections of microscopic z-stacks showing BGT 647 (“old AChR”), BGT 555 (“new AChR”), and the merge of both with BGT 647 and BGT 555 signals in green and red, respectively. **(B–D)** Quantitative analysis of fiber diameter **(B)**, ratio of BGT 555/BGT 647 signals at neuromuscular junctions (NMJs) (new AChR/old AChR) **(C)**, and number of BGT 647 puncta per NMJ **(D)** as function of PBS or 6OHD treatment. Shown is mean ± SEM, *n* = 6 muscles for PBS and *n* = 9 muscles for 6OHD. *p < 0.05, ****p < 0.00001.

As previously shown, both, chemical and surgical ablation of sympathetic neurons resulted in reduced AChR presence in membranes (Khan et al., 2016; Rodrigues et al., 2018), suggesting an altered AChR turnover under these conditions. To investigate this aspect, live imaging of mouse tibialis anterior muscles in the absence or presence of chemical sympathectomy was performed. Therefore, chemical sympathectomy was applied as previously described (Khan et al., 2016). Briefly, 6-hydroxy dopamine (6OHD) was injected into mouse hindlimb tibialis anterior for 2 weeks every other day. Similar to previous reports (Akaaboune et al., 1999; Bruneau et al., 2005; Röder et al., 2009, 2010; Choi et al., 2012; Khan et al., 2014, 2016), stability of AChR was addressed using a sequential labeling approach, where old and newly formed pools of AChR were marked with BGT 647 and BGT 555, respectively. Subsequent *in vivo* confocal analysis showed that upon sympathectomy, the relative amount of BGT 555 staining increased over that of BGT 647 (Figure 2A). Quantitative analysis confirmed a strong reduction in fiber diameter upon sympathectomy as observed previously (Khan et al., 2016; Figure 2B). Quantification of relative levels of BGT 555/BGT 647 showed an enhanced AChR turnover (Figure 2C). In addition, the number of BGT-positive puncta per NMJ was roughly doubled (Figure 2D), suggesting an increased endo/lysosomal retrieval of AChR.

### Proteomic Analysis Corroborates Regulation of Endo/Lysosomal Pathway Upon Sympathectomy

**FIGURE 3:**
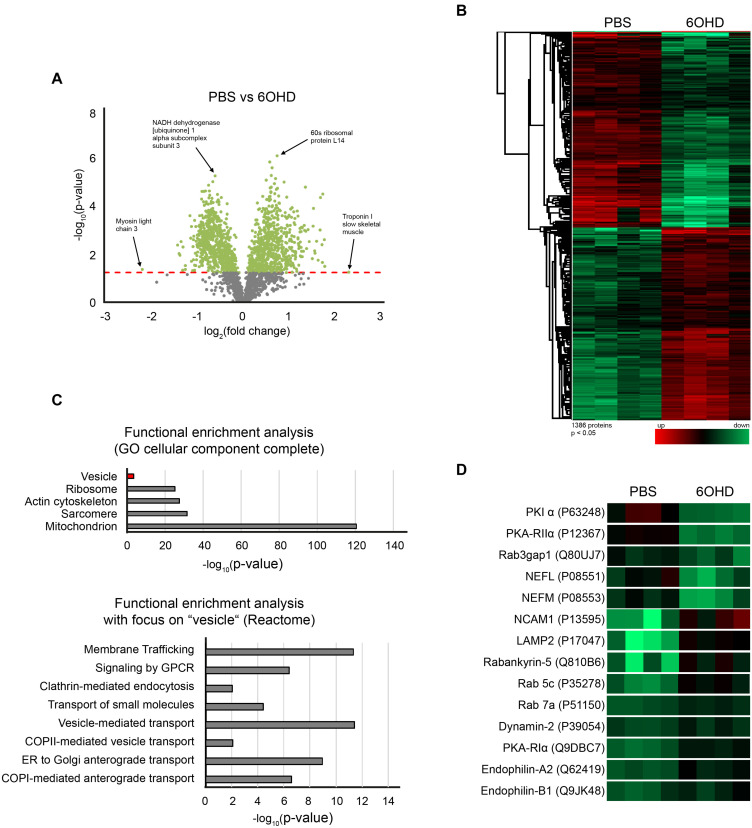
Sympathectomy induces extensive changes in mouse tibialis anterior muscle proteome. Muscles were injected with either PBS or 6OHD for 2 weeks every other day. Then, specimens were harvested and subjected to proteomic analysis. The proteomics were done on four tibialis anterior muscles per group **(A)** Volcano plot of proteomic data representing the significance (–log_10_ *p*-value) and magnitude of change (log_2_ fold change) from sympathectomized and PBS-treated tibialis anterior muscles. The red dotted line indicates the cutoff significance level of *p* < 0.05; gray and green dots represent proteins below and above this cutoff, respectively. **(B)** Heatmap of 1,386 significantly regulated proteins from sympathectomized and PBS-treated tibialis anterior muscles. **(C)** Functional enrichment analysis of proteomic data determined by gene ontology (GO) cellular component complete and Reactome databases. **(D)** Heatmap extraction of some selected proteins significantly regulated upon sympathectomy. Same color code applied as in **(B)**.

To gain wholistic and deeper mechanistic insight into the role of the sympathetic nervous system in regulating skeletal muscle physiology and potentially endocytic trafficking, proteomic analysis was performed. To study this, tibialis anterior muscles with and without chemical sympathectomy were compared. Using iTRAQ (Ross et al., 2004) technology, we could quantify 2,215 proteins (≥2 unique peptides and at ≤1% FDR) between PBS and 6OHD-treated mouse tibialis anterior samples. All mass spectrometry proteomics data have been deposited to the ProteomeXchange Consortium via the PRIDE partner repository with the dataset identifier PXD021601. In addition, they are available as Supplementary Table 1. The differential regulation of detected proteins is shown in the volcano plot and heatmap (Figures 3A,B). While Figure 3A points to a few examples of altered proteins under the categories of metabolic, ribosomal, and sarcomeric proteins, Figure 3B demonstrates a relatively homogeneous regulation profile across all tested specimens. To identify whether groups of proteins belonging to the same cellular component were regulated in a similar manner during sympathectomy, functional enrichment analysis using the “gene ontology (GO) cellular component complete” (Ashburner et al., 2000; Carbon et al., 2019) database was used. This analysis showed ample regulation of proteins of mitochondria, actin cytoskeleton, and vesicles (Figure 3C, upper diagram). Furthermore, focusing on the term “vesicle,” the “Reactome” (Fabregat et al., 2018; Jassal et al., 2020) database yielded involvement of several pathways linked to membrane trafficking, G-protein receptor signaling, and vesicle-mediated transport (Figure 3C, lower diagram).

A closer look unto individual proteins related to vesicle trafficking showed that Rab GTPases linked to endocytosis (Rab 5c, Rab 7a, Table 2 and Figure 3D) as well as regulatory proteins of Rab GTPases (Rab3gap1, Rabankyrin-5, Table 2 and Figure 3D) and lysosomal/autophagy markers (LAMP1, LAMP2, Table 2 and Figure 3D) were upregulated upon sympathectomy. The same held true for a couple of further endocytosis-related marker proteins, including Dynamin 2 (Table 2 and Figure 3D), Endophilin A2 (Table 2 and Figure 3D), and Endophilin B1 (Table 2 and Figure 3D). Protein kinase A (PKA) signaling, which was previously linked to AChR endocytosis and recycling (Röder et al., 2012, 2010), was apparently also affected by sympathectomy; indeed, while PKA regulatory subunit Iα (PKA-RIα) was found to be more abundant upon 6OHD treatment, the opposite was true for PKA-RIIα and the PKA inhibitor PKIα (Table 2 and Figure 3D).

**TABLE 2:** Extract of fold change and *p*-values of selected proteins.

UniProt Accession	Protein	6OHD/PBS	*t*-test
P63248	PKI α	0.53	0.01611*
P27573	MPZ	0.56	0.00593**
P12367	PKA-RIIα	0.57	0.00002****
P04370	MBP	0.58	0.00867**
P08551	NEFL	0.66	0.01528*
P08553	NEFM	0.68	0.01039*
Q80UJ7	Rab3gap1	0.77	0.02326*
P39054	Dynamin-2	1.13	0.04913*
P51150	Rab 7a	1.20	0.00098***
Q9JK48	Endophilin B1	1.23	0.04367*
Q62419	Endophilin A2	1.32	0.00635**
Q9DBC7	PKA-RIα	1.39	0.00015***
P11438	LAMP1	1.51	0.00183**
Q9DCD0	PGD	1.54	0.00326**
P35278	Rab 5c	1.62	0.00073***
Q810B6	Rabankyrin-5	1.86	0.01111*
P13595	NCAM1	2.18	0.00756**
P17047	LAMP2	2.34	0.00251**

Muscles were injected with either PBS or 6OHD for 2 weeks every other day. Then, samples were harvested and subjected to proteomic analysis. The table shows the fold change (6OHD/PBS) of selected proteins extracted from proteome analysis of 6OHD and PBS-treated tibialis anterior muscles. Statistical significances were determined using Student’s t-test p-values (two-tailed distribution, two-sample assuming unequal variance). *p < 0.05, **p < 0.01, ***p < 0.001, ****p < 0.0001.

Previous work by Rodrigues and colleagues reported degeneration of motoneurons and partial demyelination upon surgical sympathectomy (Rodrigues et al., 2018). Fittingly, we also found downregulation of neurofilament chains (NEFM, NEFL, Table 2 and Figure 3D) and myelin proteins (MPZ, MBP, Table 2), as well as upregulation of the protein neuronal cellular adhesion molecule (NCAM1, Table 2 and Figure 3D). Consistent with proteomic analysis of sciatic nerve denervation, which engages motor, sympathetic, and sensory axon transection (Schmalbruch, 1986; Lang et al., 2017), mitochondria-associated proteins were mostly downregulated while some cytosolic metabolic enzymes like 6 phosphogluconate dehydrogenase (PGD, Table 2) were upregulated. Finally, regulation of proteins involved in inflammation and muscle regeneration was observed (see Supplementary Figure 1). These included significant upregulation of PYCARD, involved in the inflammasome of monocytes and macrophages (Bryan et al., 2009); CFH and C3, components of the complement system, as well as ANXA1, a mediator between macrophages and muscle regeneration (McArthur et al., 2020).

### Western Blot Confirms Upregulation of Endo/Lysosomal and Autophagy Markers Upon Sympathectomy

**FIGURE 4:**
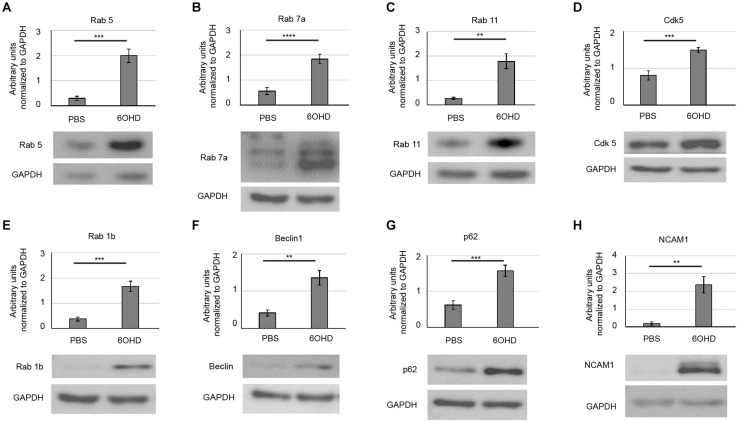
Upregulation of endo/lysosomal and autophagy markers upon sympathectomy. Muscles were injected with either PBS or 6OHD for 2 weeks every other day. Then, muscles were harvested, lysed, and subjected to Western blot analysis. Shown are representative bands of Western blots probed with antibodies for **(A)** Rab 5, **(B)** Rab 7a, **(C)** Rab 11, **(D)** Cdk5, **(E)** Rab 1b, **(F)** Beclin1, **(G)** p62, and **(H)** NCAM1. As loading control, glyceraldehyde 3-phosphate dehydrogenase (GAPDH) was used. Bar graphs depict protein band intensities relative to their corresponding GAPDH band [**(A,B,D,G)** mean ± SEM; *n* = 7 muscles for each, **(C,E,F,H)** mean ± STD; *n* = 7 muscles for each. **p < 0.01, ***p < 0.001, ****p < 0.0001).

The present proteomic data (Rab 5c, Rab 7a, LAMP1/2, Endophilin A2, Endophilin B1), previously described shrinkage of NMJ size (Khan et al., 2016), and the reduced AChR presence at the plasma membrane (Rodrigues et al., 2018) upon sympathectomy argued for an upregulated turnover and endocytic retrieval of AChR. To confirm these findings, we next tested the effect of sympathectomy on the abundance of several markers of the endo/lysosomal and autophagic pathways by Western blot analysis. This revealed upregulation upon sympathectomy of Rab proteins relevant for early endosomes (Rab 5, Figure 4A), late endosomes (Rab 7a, Figure 4B), and recycling endosomes (Rab 11, Figure 4C). Since regulation of Rab 5 was shown to involve Cdk5, its protein levels were also analyzed and found to be elevated upon 6OHD treatment (Figure 4D). Furthermore, markers for autophagy were increased upon sympathectomy, including Rab 1b, which is important for autophagosome formation from the ER (Figure 4E), Beclin1, an essential component of autophagy initiation (Figure 4F), and p62 (aka SQSTM1) (Figure 4G), a ubiquitin-binding protein also known to be involved in AChR turnover (Khan et al., 2014). Finally, NCAM1 was upregulated upon sympathectomy (Figure 4H).

### Endplate Acetylcholine Receptor-Staining Intensity Is Reduced at Neuromascular Junctions Upon Sympathectomy

**FIGURE 5:**
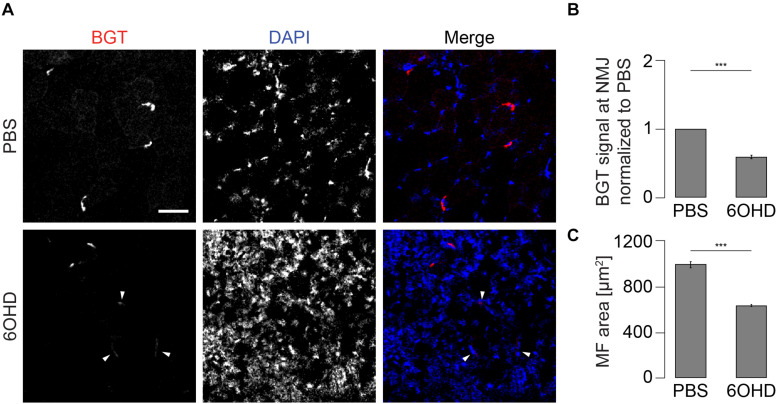
AChR density at NMJs is reduced in 6OHD-treated muscles. Tibialis anterior muscles were injected with either PBS or 6OHD for 2 weeks every other day. Then, specimens were harvested, sectioned, and stained with BGT and DAPI for AChR and nuclei, respectively. **(A)** Panels depict representative confocal images of fluorescence signals as indicated. In merge panels, NMJs and nuclei are shown in red and blue, respectively. Exemplary faint NMJs in the presence of 6OHD are indicated by arrowheads. Scale bar, 50 μm. Graphs show quantitative analysis of average BGT intensity at NMJs **(B)** and of average muscle fiber (MF) area **(C)**. Depicted is mean ± SEM, ***p < 0.001, *n* = 5 muscles.

Given the observed increase in endo/lysosomal trafficking of AChR-containing vesicles (see Figure 2), we wanted to address the consequences of this increased turnover on the AChR intensity at the NMJ. For this purpose, the live imaging data in Figure 2 were inappropriate because in these experiments, the imaging and display settings were adjusted to optimize for the ratiometric analysis and involved tissue depths of a few hundred microns. Thus, we opted here for a more consistent approach for measuring AChR staining intensity, i.e., on tissue sections. These were prepared of muscles treated with either PBS or 6OHD and then stained with BGT coupled to a fluorescent dye. Fluorescence intensity was measured at the NMJ. As shown in Figures 5A,B, BGT intensity was reduced upon 6OHD treatment, and 6OHD-treated muscle sections were characterized by weakly stained NMJs (for exemplary weakly stained NMJs, see arrowheads in Figure 5A). The observed increase in nuclei density (DAPI, blue) fitted to the upregulated inflammatory response and muscle regeneration (see Supplementary Figure 1) as well as to the observed reduction of muscle fiber area of approximately 35% (Figure 5C).

### p62 and Rab 7a Intensities Are Increased at Neuromascular Junctions Upon Sympathectomy

**FIGURE 6:**
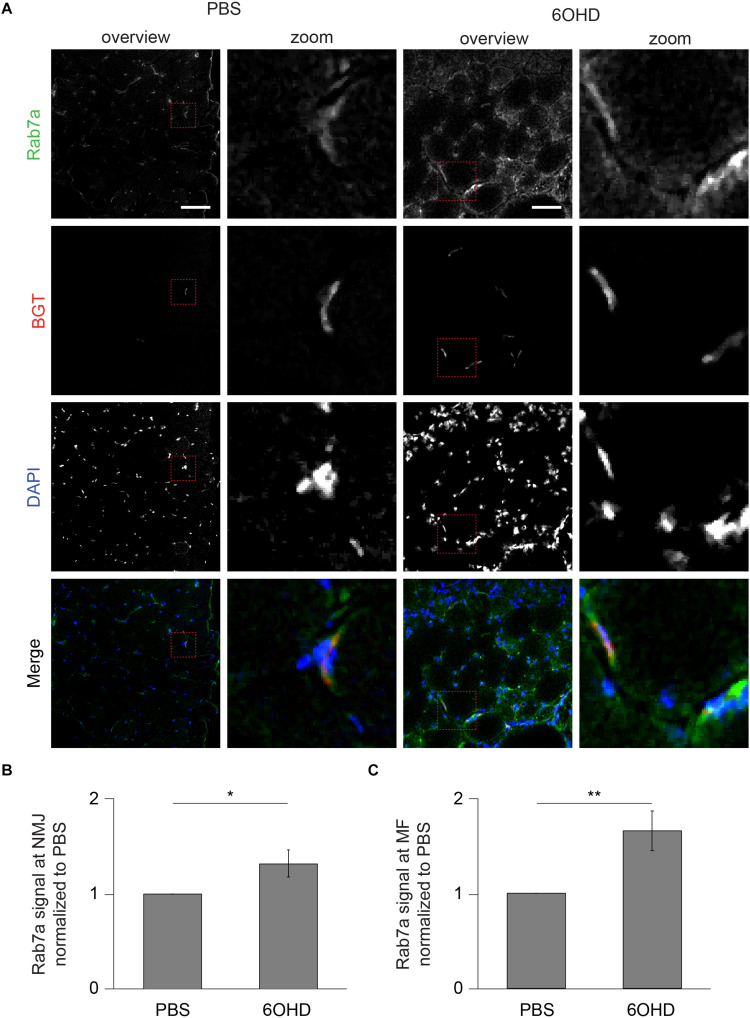
Upon sympathectomy, Rab 7a intensity is increased in muscle fibers and at NMJs. Tibialis anterior muscles were injected with either PBS or 6OHD for 2 weeks every other day. Then, specimens were harvested, sectioned, and stained with anti-Rab 7a antibody, BGT, and DAPI for Rab 7a, AChR, and nuclei, respectively. **(A)** Panels depict representative confocal images of fluorescence signals as indicated. In merged panels, Rab 7a, NMJs, and nuclei are shown in green, red, and blue, respectively. Detail pictures with representative NMJ regions, indicated with dashed lines in “overview” picture columns, are shown in “Zoom” columns. Scale bars, 50 μm. Graphs show quantitative analysis of average Rab 7a signal intensity at **(B)** NMJs and **(C)** muscle fibers (MF). Depicted is mean ± SEM (*p < 0.05, **p < 0.01, *n* = 5 muscles).

**FIGURE 7:**
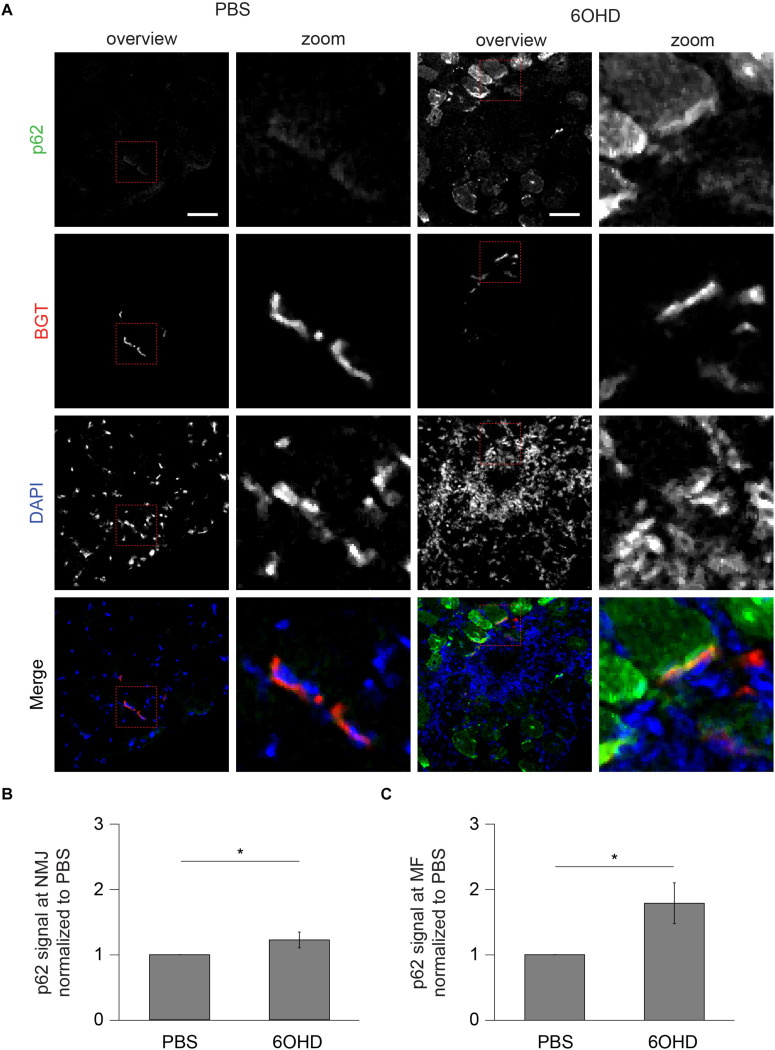
Upon sympathectomy, p62 intensity is increased in muscle fibers and at NMJs. Tibialis anterior muscles were injected with either PBS or 6OHD for 2 weeks every other day. Then, specimens were harvested, sectioned, and stained with anti-p62 antibody, BGT, and DAPI for p62, AChR, and nuclei, respectively. **(A)** Panels depict representative confocal images of fluorescence signals as indicated. In merged panels, p62, NMJs, and nuclei are shown in green, red, and blue, respectively. Detail pictures with representative NMJ regions, indicated with dashed lines in “overview” picture columns, are shown in “Zoom” columns. Scale bars, 50 μm. Graphs show quantitative analysis of average p62 signal intensity at **(B)** NMJs and **(C)** muscle fibers (MF). Depicted is mean ± SEM (*p < 0.05, *n* = 5 muscles).

**FIGURE 8:**
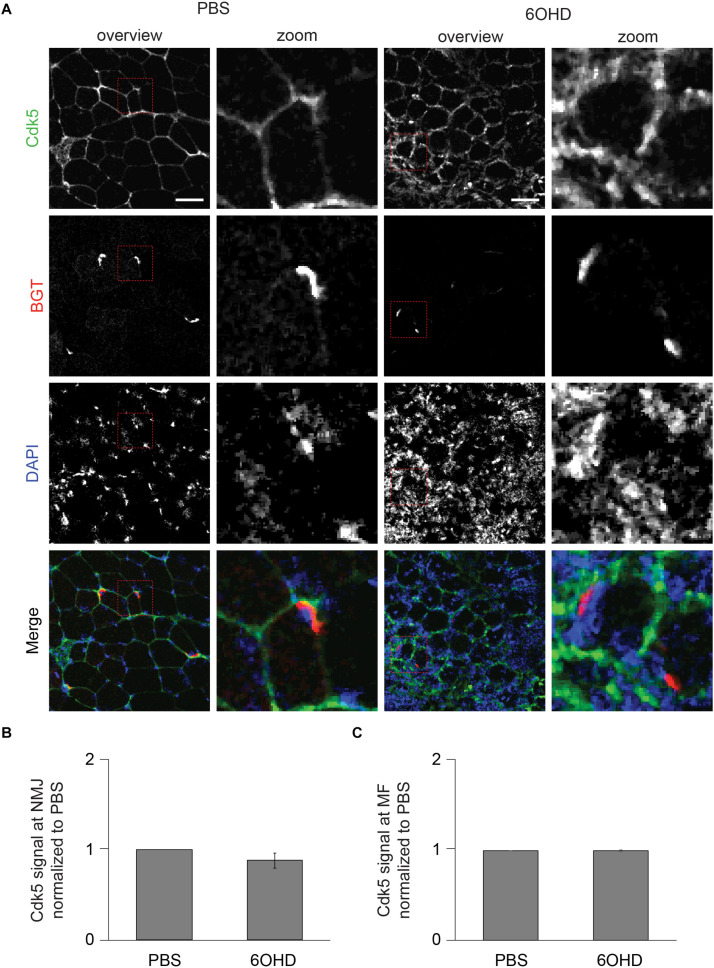
Upon sympathectomy, Cdk5 intensity is unaltered in muscle fibers and at NMJs. Tibialis anterior muscles were injected with either PBS or 6OHD for 2 weeks every other day. Then, specimens were harvested, sectioned, and stained with anti-Cdk5 antibody, BGT, and DAPI for Cdk5, AChR, and nuclei, respectively. **(A)** Panels depict representative confocal images of fluorescence signals as indicated. In merged panels, Cdk5, NMJs, and nuclei are shown in green, red, and blue, respectively. Detail pictures with representative NMJ regions, indicated with dashed lines in “overview” picture columns, are shown in “Zoom” columns. Scale bars, 50 μm. Graphs show quantitative analysis of average Cdk5 signal intensity at **(B)** NMJs and **(C)** muscle fibers (MF), mean ± SEM (*n* = 5 muscles). Not significantly different.

Since proteomic and Western blot analysis lack spatial resolution, localizations of representative, upregulated proteins were analyzed on muscle slices by means of immunofluorescence staining and subsequent confocal microscopy. Slices were stained with BGT for AChR, with DAPI for nuclei, and with antibodies against either Rab 7a, p62, or Cdk5. Under basal conditions, all three markers were slightly enriched at the NMJs but with low intensity within the muscle fibers (Figures 6–8). In addition, Cdk5 was present along the entire sarcolemma (Figure 8). Upon sympathectomy, upregulation of Rab 7a (Figures 6A–C) and p62 (Figures 7A–C) staining at the NMJ as well as in the muscle fiber was observed. Conversely, Cdk5 levels remained unchanged at both these sites (Figure 8), but an increase was observed in the sarcolemma outside the NMJ area and in the interstitial space, potentially indicating the presence of immune cells (Shupp et al., 2017) or of neurodegenerative and regenerative processes (Hwang and Namgung, 2020).

## Discussion

Recent work has revealed innervation of NMJs by sympathetic neurons (Khan et al., 2016; Rodrigues et al., 2018, 2019; Straka et al., 2018; Snyder-Warwick et al., 2018). This is important for morphological and functional maintenance of NMJs (Khan et al., 2016; Rodrigues et al., 2018, 2019) and has served as a plausible explanation for the beneficial effect of sympathicomimetics in the treatment of several forms of myasthenic syndromes (Lee et al., 2018; Finsterer, 2019; Thompson et al., 2019). Here, we investigated whether sympathetic innervation might act on endo/lysosomal trafficking at the NMJ by means of *in vivo* confocal microscopy, proteomic, Western blot, and immunofluorescence analyses of muscles treated with or without chemical sympathectomy. Our studies revealed an increase in AChR turnover and upregulation of endo/lysosomal AChR vesicles as well as autophagic marker proteins in sympathectomized samples, suggesting a regulatory function of sympathetic input on these pathways.

Endo/lysosomal trafficking of AChR-containing vesicles requires a highly orchestrated interaction of various proteins and cAMP-signaling, which can be induced by sympathetic stimuli (Rudolf and Straka, 2019). Recent transcriptome analysis (Rodrigues et al., 2018) and the data presented here reveal an altered abundance of a variety of proteins that are linked to endo/lysosomal trafficking and autophagy. As for the first, Western blot analysis confirmed upregulation of Rab 5 and Rab 7a. With all warranted caution with respect to quantitative immunofluorescence analysis, this suggested enhanced Rab 7a levels both at the NMJs and also in the cytosol. Thus, it appears that the increase upon sympathectomy of Rab 7a was not NMJ specific, but might have affected the entire muscle fiber. For Rab 5, immunofluorescence signals were not specific enough to permit such conclusions (data not shown). Previous work on *in vivo* imaging of Rab 5-transfected muscles demonstrated colocalization of AChR-containing vesicles with Rab 5 fluorescence and transfection of a constantly active Rab 5Q79L mutant led to a strong increase in endocytic BGT-positive endosomes (Wild et al., 2016). With respect to autophagy markers, Western blot or proteomic analysis showed that Rab 1b, p62, Beclin1, and LAMP1/2 were enhanced upon sympathectomy. Among these markers, p62 was also studied by immunofluorescence. Similar to Rab 7a, this suggested the upregulation of p62 signals both within the sarcomeric region of muscle fibers as well as at NMJs. This might indicate that sympathetic neuronal signaling plays a crucial role in autophagic degradation of muscle tissue, and it is consistent with a role in the decay of AChR, as shown previously (Khan et al., 2014).

In contrast to Rab 1b, Rab 5, Rab 7a, p62, Beclin1, and LAMP1/2, the increase in Rab 11 and Cdk5 as observed upon proteomics and Western blot was less expected. To start with Rab 11, this is well-known for its role in regulating vesicle recycling (Lock and Stow, 2005; Takahashi et al., 2012; Welz et al., 2014; Redpath et al., 2020) and was shown to be important also for AChR recycling (Wild et al., 2019). Therefore, its upregulation upon sympathectomy is intriguing. An explanation for this observation might be based on the additional roles of Rab 11 in orchestrating multivesicular bodies, autophagy (Longatti et al., 2012; Fader et al., 2008; Szatmári et al., 2014), and autophagosome biogenesis (Puri et al., 2018). Furthermore, studies in a hepatocarcinoma cell line suggests requirement of cAMP for Rab 11 activity (Won Park et al., 2014). Whether reduced cAMP signaling by sympathectomy causes accumulation of Rab 11-positive vesicles due to reduced Rab 11 activity needs further investigation. Next, for Cdk5, we previously showed that transfection with a dominant-negative Cdk5 blunted the typical denervation-induced increase in AChR-containing vesicles (Wild et al., 2016). Therefore, it was speculated whether a block of Cdk5 activity correlated with increased endocytic processing of AChR. While the enhanced total level of Cdk5 upon sympathectomy observed here does not accord with this assumption, our immunofluorescence analysis in 6OHD-treated cryosections revealed no specific increase in Cdk5 signal, neither in the sarcomeric regions nor at the NMJ. However, Cdk5 immunofluorescence was apparently enhanced in the sarcolemma adjacent to NMJs and also in the space in between muscle fibers. Given the well-known function of Cdk5 in fast adaptation of the cytoskeleton in neurons (Shah and Rossie, 2018), a general increase in Cdk5 in the sarcolemma might be a response to the described atrophy in sympathectomized muscles (Khan et al., 2016). In addition, Cdk5 is known to be upregulated in activated immune cells (Shupp et al., 2017) and upon neurodegeneration and regeneration (Hwang and Namgung, 2020). In combination with our proteomics and DAPI staining data, which showed an increase in markers related to immune cells and in density of nuclei upon sympathectomy, the increased Cdk5 levels might have been partly due to the immune and de/regeneration processes going on after 6OHD treatment. Furthermore, the sheer presence of Cdk5 does not necessarily correlate to its activity, which additionally requires its association with a regulatory subunit, p35 (Patrick et al., 1999). On another note, higher levels of Cdk5 were reflected by an upregulation upon sympathectomy of Cdc42 in proteomics. Indeed, Cdk5 indirectly activates Cdc42 via TrkB phosphorylation (Cheung et al., 2007). Cdc42 is a small GTPase of the Rho family that mediates agrin-induced AChR clustering together with Rac1 (Weston et al., 2000) and might serve as a regulator in clathrin-independent endocytosis together with endophilins (Sabharanjak et al., 2002; Howes et al., 2010; Sandvig et al., 2018; Redpath et al., 2020). Rac1 (not regulated, Supplementary Table 1), another small GTPase of the Rac family, is, besides its role in AChR clustering (Weston et al., 2000), known to be inhibited by RCC2 (upregulated, Supplementary Table 1; Tilley et al., 2015) and to interact with coronin 1C (upregulated, Supplementary Table 1). Generally, coronins are actin-associated proteins involved in remodeling of the cytoskeleton (De Hostos et al., 1991; De Hostos, 1999; Tilley et al., 2015). So far, three types of coronins, all altered here in abundance upon sympathectomy (Supplementary Table 1), have been classified based on sequence homology: type I includes coronins 1 A–C as well as coronin 6, type II is represented by coronins 2A and B, and finally, coronin 7 belongs to type III (Chen et al., 2014). Association of coronins with the actin-related protein 2/3 (Arp2/3) complex (Arpc2, Arpc3, both upregulated, Supplementary Table 1) was suggested based on experiments in yeast (Humphries et al., 2002). Further analysis showed regulation of coronin 1B (upregulated, Supplementary Table 1) and the Arp2/3 complex via protein kinase C (Cai et al., 2005). Coronin 6 (downregulated, Supplementary Table 1) and coronin 1C are the main isoforms expressed in healthy skeletal muscle, and destabilization of AChR was found *in vivo* upon reduction of coronin 6 (Chen et al., 2014). Potentially, this was due to disturbances in interaction of AChR and actin (Chen et al., 2014). Whether the sympathectomy-induced regulation of markers acting on the actin cytoskeleton observed in the proteomics analysis is specific to NMJs has still to be determined. Since cytoskeletal rearrangements are also needed for vesicle trafficking, the observation of increased endo/lysosomal trafficking, which also affects AChR turnover, might be correlated with this.

Changes in AChR turnover are a hallmark of aging (Gonzalez-Freire et al., 2014; Rudolf et al., 2014; Taetzsch and Valdez, 2018). Interestingly, enhanced presence of TH staining at the NMJs of a mouse model with brain-specific overexpression of Sirt1 (Satoh et al., 2013) correlated with slowed aging and “healthier-looking” NMJs compared with age-matched wild types (Snyder-Warwick et al., 2018). Along these lines, a Troponin T (cardiac)-promoted switch between PKA regulatory subunit type Iα (RIα) and PKA-RIIα/RIIβ at the NMJ was previously reported upon aging (Xu et al., 2017). In the present study, we found an increase in AChR turnover upon sympathectomy that was accompanied by enhanced PKA-RIα levels together with a reduction in PKA-RIIα, similar to that described by Rodrigues et al. (2018). This suggests that lowered sympathetic input might induce a switch of PKA regulatory subunits. Although only PKA-RIα, but not PKA RIIα/RIIβ, was found to be associated with AChRs (Röder et al., 2010), this might change in old age or upon sympathectomy.

In addition to the effects of sympathectomy on skeletal muscle, recent work (Rodrigues et al., 2018) using surgical sympathectomy described also motoneuronal neurofilament dephosphorylation and a reduced presynaptic synaptophysin staining (Rodrigues et al., 2018). That study used microsurgical bilateral excision of the second and third lumbar (L2-L3) ganglia of the paravertebral sympathetic chain and sample harvest 7 days later (Rodrigues et al., 2018). This procedure yielded an upregulation of transcripts related to denervation such as *chrng* and *myogenin* (Rodrigues et al., 2018), but no increase in NCAM mRNA, although this is considered as a solid sign for denervation (Covault and Sanes, 1985; Rodrigues et al., 2018). Conversely, we found upregulation of NCAM protein levels, both, in the proteomics and the Western blot analyses. This discrepancy might be explained by a finding of Lang et al., where a denervation-mediated rise of NCAM protein levels was not due to increased NCAM protein synthesis but rather enhanced NCAM protein stability (Lang et al., 2017).

To sum up, the present data are consistent with a previously suggested model where sympathetic neurons not only innervate blood vessels but also motoneurons, muscle fibers, and NMJs (Khan et al., 2016). It appears that sympathectomy has a major impact on metabolism and protein trafficking. In particular, while mitochondrial markers were massively turned down, endo/lysosomal, and autophagic pathways were enhanced.

## Data Availability Statement

The datasets presented in this study can be found in online repositories. The names of the repository/repositories and accession number(s) can be found in the article/Supplementary Material.

## Ethics Statement

The animal study was reviewed and approved by Regierungspräsidium Karlsruhe Schlossplatz 4–6 76131 Karlsruhe.

## Author Contributions

AR and RR conceptualized the study and were in charge of the project administration and supervision. TS, CS, LK, MW, and MK were in charge of the methodology. TS, AR, and RR did the validation and reviewed, edited, and wrote the manuscript. TS, CS, LK, and MK performed the formal analysis. TS and MK conducted the investigation. AR, AS, MH, and RR were responsible for the collection of resources and were responsible for the funding acquisition. TS and AR handled the data curation and made the visualization of the study. TS prepared and wrote the original draft. All authors have read and agreed to the published version of the manuscript.

## Conflict of Interest

The authors declare that the research was conducted in the absence of any commercial or financial relationships that could be construed as a potential conflict of interest.

## Footnotes

1 https://maxquant.net/perseus/

2 https://imagej.nih.gov/ij/

## Supplementary Material

Supplementary Figure 1 Sympathectomy induces regulation of inflammatory response and skeletal muscle regeneration. Muscles were injected with either PBS or 6OHD for 2 weeks every other day. Then, specimens were harvested and subjected to proteomic analysis. The proteomics were done on four tibialis anterior muscles per group. Functional enrichment analysis of proteomic data determined by GO database for “Inflammatory response” (upper panel) and “Skeletal muscle regeneration” (lower panel). Asterisks mark proteins significantly regulated with *p* ≤ 0.05.
