# Supplementary material for: Regulatory Function of Sympathetic Innervation on the Endo/Lysosomal Trafficking of Acetylcholine Receptor
Source: Front Physiol. 2021 Mar 11;12:626707. doi: 10.3389/fphys.2021.626707 (PMC7991846; doi:10.3389/fphys.2021.626707)
Supplement: Supplementary Figure 1 — Sympathectomy induces regulation of inflammatory response and skeletal muscle regeneration. Muscles were injected with either PBS or 6OHD for 2 weeks every other day. Then, specimens were harvested and subjected to proteomic analysis. The proteomics were done on four tibialis anterior muscles per group. Functional enrichment analysis of proteomic data determined by GO database for “Inflammatory response” (upper panel) and “Skeletal muscle regeneration” (lower panel). Asterisks mark proteins significantly regulated with p ≤ 0.05. [file Image_1.pdf]

Supplementary Figure 1

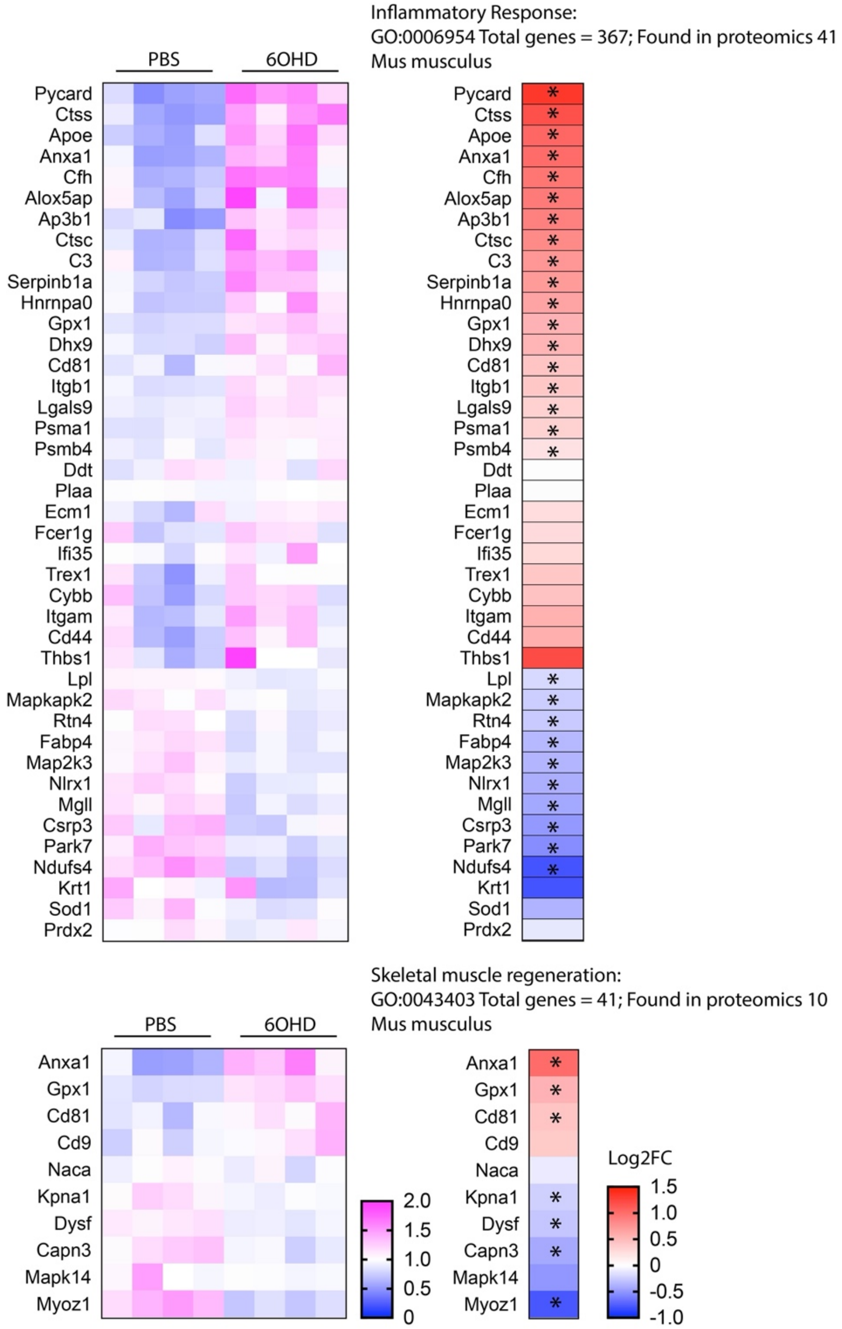

Figure S1. Sympathectomy induces regulation of inflammatory response and skeletal muscle regeneration. *Muscles were injected with either PBS or 6OHD for 2 weeks every other day. Then, specimens were harvested and subjected to proteomic analysis. The proteomics were done on four tibialis anterior muscles per group. Functional enrichment analysis of proteomic data determined by GO database for "Inflammatory response" (upper panel) and "Skeletal muscle regeneration" (lower panel). Asterisks mark proteins significantly regulated with p≤ 0.05.*
