# Supplementary material for: Regulatory Function of Sympathetic Innervation on the Endo/Lysosomal Trafficking of Acetylcholine Receptor
Source: Front Physiol. 2021 Mar 11;12:626707. doi: 10.3389/fphys.2021.626707 (PMC7991846; doi:10.3389/fphys.2021.626707)
Supplement: Supplementary file 2 [file Data_Sheet_1.docx]

Supplementary Material

# Protocol for Proteomic Analysis

**Materials**

Following materials were purchased from Sigma-Aldrich, Germany: ammonium acetate, ammonium bicarbonate (NH_4_HCO_3_), ammonium hydroxide (NH_4_OH) solution (≥25% in H_2_O), anhydrous magnesium chloride (MgCl_2_), guanidine hydrochloride (GuHCl), iodoacetamide (IAA), triethylammonium bicarbonate (TEAB), and urea. Tris base was bought from Applichem Biochemica, Darmstadt, Germany. Sodium dodecyl sulfate (SDS) was purchased from Carl Roth, Karlsruhe, Germany. Dithiothreitol (DTT), complete mini EDTA-free (protease inhibitor cocktail tablets) was bought from Roche Diagnostics, Mannheim, Germany. Sodium chloride (NaCl) and calcium chloride (CaCl_2_) were purchased from Merck, Darmstadt. Benzonase Nuclease was purchased from Novagen. Sequencing grade modified trypsin was purchased from Promega, Madison, USA. Bicinchoninic acid assay (BCA) kit was bought from Pierce Thermo Fisher Scientific, Schwerte, Germany. Isobaric tags for relative and absolute quantification (iTRAQ) 8plex reagents kit (113-119, 121) was purchased from AB SCIEX, Framingham, USA. All chemicals for ultra-pure HPLC solvents such as formic acid (FA), trifluoroacetic acid (TFA) and acetonitrile (ACN) were purchased from Biosolve, Valkenswaard, the Netherlands.

**Methods**

***Tissue lysis, carbamidomethylation and on-filter proteolysis***

Tibialis anterior muscles (right and left) derived from four different mice treated either with phosphate buffer saline (PBS) or with 6-hydroxydopamine (6OHD) were used for proteomics analysis. The tissue sections were first snap frozen using liquid nitrogen followed by manual grinding with a metal dounce. Samples were then lysed with 1% SDS buffer containing 50 mM Tris-Cl, 150 mM NaCl, pH 7.8 with complete mini. To degrade nucleic acids, tissue lysates were treated with Benzonase and 2 mM MgCl_2_ and incubation at 37°C for 30 min. The lysates were centrifuged at 18,000 rcf at room temperature (RT) for 30 min. The clear supernatant was collected and used to determine protein concentration with BCA assay as per manufacturer’s instructions. Samples corresponding to ~150 µg of protein of each sample were subjected to carbamidomethylation i.e. reduction of disulfide bonds with 10 mM DTT and incubation at 56°C for 30 min followed by alkylation of free thiol groups with 30 mM IAA and incubation at RT for 30 min in the dark. Next, sample cleaning and on-filter proteolytic digestion (trypsin) were performed using the filter aided sample preparation [1, 2] protocol with nanosep centrifugal devices (30 KDa, PALL), but with minor changes as previously described [3]. Tryptic peptides were quality controlled [4] and were desalted with C18 solid phase extraction cartridges (SPEC; 100 µL, Varian) according to the manufacturer’s instructions and the eluted peptides were dried in a SpeedVac and stored at -80°C until further use.

***iTRAQ labeling and high pH (8.0) C18 reversed-phase fractionation***

The dried peptides were resolubilized in 30 µL of 0.5 M TEAB buffer, pH 8.5 and samples were labeled with iTRAQ reagents [5] according to the manufacturer’s instructions. After labeling and incubation at RT for 2 h, 1 µL of each sample was pooled and analyzed by LC-MS to evaluate labeling efficiency and to correct minor differences of sample amounts e.g. due to pipetting errors. Afterwards, samples were mixed in 1:1:1:1:1:1:1:1 ratio based on the normalization factors obtained from the prior LC-MS analysis. An aliquot corresponding to ~20 µg of the multiplexed sample was desalted with C18 SPEC 100 µL tips. Eluted peptides were dried in a SpeedVac and resolubilized in 10 mM NH_4_OH, 17 mM FA, pH 8.0 (Buffer A) and fractionated on a C18 reversed-phase column at pH 8.0 as described earlier [3]. In total 16 fractions were collected at 1 min intervals from min 5 to 70 in a concatenation mode. The fractions were completely dried in a SpeedVac and stored at -80°C until further use.

***LC-MS/MS analysis***

Each individual high-pH (8.0) fraction (N = 16) was resolubilzed in 0.1% TFA and only 50% of each sample was analyzed using an Ultimate 3000 nano RSLC system coupled to a Q Exactive HF mass spectrometer (both Thermo Scientific, Germany). Peptides were preconcentrated on a 100 µm x 2 cm C18 trapping column for 5 min using 0.1% TFA with a flow rate of 20 µL/min followed by separation on a 75 µm x 50 cm C18 main column (both Acclaim Pepmap nanoviper, Thermo Scientific, Germany) with 120 min LC gradient ranging from 3-35% of B (84% ACN in 0.1% FA) at a flow rate of 250 nL/min. The Q Exactive HF was operated in data-dependent acquisition mode and MS survey scans were acquired from m/z 300 to 1500 at a resolution of 60000 using the polysiloxane ion at m/z 371.1012 as lock mass [6]. The twenty most intense ions were isolated with a 0.4 m/z window and fragmented by higher energy collisional dissociation with a normalized collision energy of 33%, taking into account a dynamic exclusion of 30 s. MS/MS spectra were acquired at a resolution of 15000. Automatic gain control target values and fill times were set to 3 × 10^6^ and 50 ms for MS and 2 × 10^5^ and 200 ms for MS/MS, respectively. Furthermore, a 10% (v/v) NH_4_OH solution was placed in front of the ESI source for charge state reduction [7].

***iTRAQ data analysis and interpretation***

All iTRAQ data (16 raw files) were processed simultaneously using the MudPIT option with Proteome Discoverer 1.4 (Thermo Scientific, Germany) and searched in a target/decoy approach against the mouse Uniprot database (with 16802 target entries, downloaded in July 2016) using three different search algorithms i.e. Mascot (Matrix Science), Sequest and MS Amanda using same set of parameters as following. Mass tolerances were set to 10 ppm and 0.02 Da for MS and MS/MS, respectively. Trypsin was selected as enzyme with a maximum of two missed cleavages; carbamidomethylation of Cys (57.0214 Da) and iTRAQ-8-plex on N terminus and Lys (304.2053 Da) were set as fixed modifications whereas, oxidation of Met (15.9949 Da) as variable modifications. The vendor provided isotope purity correction factors were incorporated in the Reporter ion node. Percolator node was included to estimate the false discovery rate (FDR). Data export was done using following filter criteria: peptide-spectrum matches with FDR ≤ 1% (high confidence setting), search engine rank 1 and only proteins that were quantified with ≥ 2 unique peptides were considered for further data analysis. Next, the normalization of the raw iTRAQ ratios was done using Excel (Microsoft) as described before [3] to get normalized abundance values (NAVs) for each protein. The NAVs of the corresponding channels were averaged accordingly and the ratios were calculated between 6OHD/PBS for each protein. Lastly, Student's t-Test p-values (two-tailed distribution, two-sample assuming unequal variance) were calculated for each protein.

1. Manza, L.L., et al., *Sample preparation and digestion for proteomic analyses using spin filters.* PROTEOMICS, 2005. **5**(7): p. 1742-1745.

2. Wisniewski, J.R., et al., *Universal sample preparation method for proteome analysis.* Nat Meth, 2009. **6**(5): p. 359-362.

3. Shema, G., et al., *Simple, scalable, and ultrasensitive tip-based identification of protease substrates.* Molecular &amp;amp; Cellular Proteomics, 2018. **17**(4): p. 826.

4. Burkhart, J.M., et al., *Systematic and quantitative comparison of digest efficiency and specificity reveals the impact of trypsin quality on MS-based proteomics.* Journal of Proteomics, 2012. **75**(4): p. 1454-1462.

5. Ross, P.L., et al., *Multiplexed Protein Quantitation in Saccharomyces cerevisiae Using Amine-reactive Isobaric Tagging Reagents.* Molecular & Cellular Proteomics, 2004. **3**(12): p. 1154-1169.

6. Olsen, J.V., et al., *Parts per Million Mass Accuracy on an Orbitrap Mass Spectrometer via Lock Mass Injection into a C-trap.* Molecular & Cellular Proteomics, 2005. **4**(12): p. 2010-2021.

7. Thingholm, T.E., et al., *Undesirable Charge-Enhancement of Isobaric Tagged Phosphopeptides Leads to Reduced Identification Efficiency.* Journal of Proteome Research, 2010. **9**(8): p. 4045-4052.
